# ASSOCIATING LIVER RADIOFREQUENCY AND PORTAL VEIN LIGATION FOR STAGED HEPATECTOMY

**DOI:** 10.1590/S0102-67202015000300019

**Published:** 2015

**Authors:** Fábio Luiz WAECHTER, Rinaldo Danesi PINTO, Felipe KOLESKI, José Artur SAMPAIO, Uirá Fernandes TEIXEIRA

**Affiliations:** 1 Surgery Unit, Federal University of Health Sciences of Porto Alegre, Santa Casa de Misericórdia de Porto Alegre, RS; 2 Digestive Surgery Unit, Regional University of Blumenau, Santa Catarina Hospital, Blumenau, SC, Brazil

## INTRODUCTION

We read with special interest the article by Schnitzbauer et al. 4 published on March 2012. We believe that this paper is a cornerstone in hepatic surgery, bringing a new method which can greatly contribute do increase resectability in patients once outside of surgical therapy.

Surgical resection remains the treatment of choice for patients with primary and secondary liver tumors, representing the only chance to obtain long-term survival 1 . Nowadays, with improvements in surgical expertise, anesthesia and postoperative care, no limits due to number of lesions and location are of value as in the past 5 .

 Since the original cited report, some technical changes in ALPPS procedure (Associating Liver Partition and Portal vein ligation for Staged Hepatectomy) were described. Despite the initial enthusiasm with the new technique, several centers worldwide showed that, when properly indicated, the morbidity related mainly to the first surgery is high 2 . The release of hepatic ligaments and the transection of the liver parenchyma when the division of segments III and IV is often responsible for increased blood loss, biliary fistula and high operative time. 

Thus, based on our previous experience with the use of bipolar radiofrequency with cold needles (BRCN) in performing hepatectomies 3 , coupled with our enthusiasm with this new two-staged technique, we decided to replace the hepatic parenchyma transection by making two lines of denatured liver tissue by radiofrequency, isolating the future liver remnant (FLR) in a similar way of surgical transection, more quickly, easily, with no hepatic mobilization and less blood loss. 

This is a report of an initial experience, which we call ALRPS - associating liver radiofrequency and portal vein ligation for staged hepatectomy.

## CASE REPORT

**FIGURE 1:**
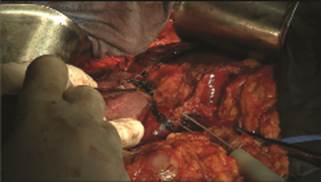
Columns of denatured liver tissue made by radiofrequency

We performed the procedure in a 62-year-old woman with colorectal liver metastasis affecting the right liver and segment IV, without extrahepatic disease. Preoperative hepatic volumetry estimated FLR of 180 cm³. In the first surgery, liver lobes were separated without hepatotomy or hepatic mobilization, only with two lines of denatured liver tissue made by BRCN ( Figure 1 ). We did not use plastic bag; instead, we covered the liver with a bioresorbable membrane to protect it. The right portal vein was ligated, it was performed ablation of middle hepatic vein and a tubular drain was placed. No blood transfusion was required.

**FIGURE 2:**
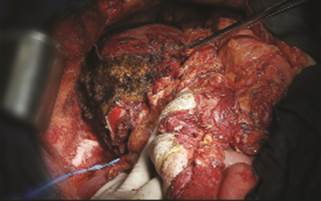
Completion of extended right hepatectomy

After 20 days, a CT volumetry showed that the left lateral liver lobe had increased to 464 cm³ approximately, a surprising hypertrophy of about 158%. Relaparotomy was scheduled for the following day, with completion of an extended right hepatectomy ( Figure 2 ). The postoperative course was uneventful.

## DISCUSSION

With the advent of ALPPS, it became possible to achieve hypertrophy of FLR at 75% on average, in a quick way and over a mean period of nine days 4 . However, the authors reported high morbidity and mortality rates, particularly with regard to the initial surgery, with high rates of biliary fistula and intraoperative bleeding.

In our case, with the use of BRCN, there is no need for extensive hepatic mobilization. Thus, it is possible to perform the first procedure with a smaller abdominal incision. By making two columns of denatured liver tissue we eliminate the collateral branches between segments III and IV, with excellent results in the remnant liver hypertrophy (158%). Furthermore, the occurrence of biliary fistula reduces significantly and, in the second surgery, the liver parenchyma can be cut with a scalpel in a quick and simple bloodless way.

We believe that necrosis induced by radiofrequency is a strong metabolic stimulus for migration of angiogenic factors and liver regeneration, adding an important contribution for FLR hypertrophy, since the increase in our report was far above the average of the initial work. It is not possible to draw conclusions from a single report. Further studies are needed, and is already underway our case series. 

Thus, using BRCN in two stage hepatectomies represents a new technique to facilitate the procedure. Its use in conjunction with portal ligation, which we named ALRPS procedure, is easy to perform and has its own advantages, especially with regard to the reduction of surgical trauma of a complex hepatotomy and its complications (perioperative bleeding, prolonged surgical time), as well as obviate the dissection of hepatic ligaments.
